# Mental Health Help-Seeking by US College Students with a Diagnosis of Psychosis

**DOI:** 10.21203/rs.3.rs-7435461/v1

**Published:** 2025-09-05

**Authors:** Clara Godoy-Henderson, Sara (Shiv) Denner, Michelle Flesaker, Janice Weinberg, Melissa Dalhoe, Sarah K Lipson

**Affiliations:** University of Minnesota; Boston University; Boston University; Boston University; University of Minnesota; Boston University

**Keywords:** psychosis, antipsychotic medication, therapy/counseling, informal support, help-seeking behaviors

## Abstract

**Purpose:**

This study aims to examine the perceptions, beliefs, and attitudes that affect help-seeking behaviors among college students with a diagnosis of psychosis.

**Methods:**

Cross-sectional 2015–2024 national survey data from the Healthy Minds Study (HMS) were used to examine mental health service utilization among 2,819 U.S. college students with a diagnosis of psychosis. Descriptive statistics and adjusted logistic regression models were used to examine students’ help-seeking behaviors.

**Results:**

Approximately eight-in-ten students with a diagnosis of psychosis reported utilizing formal services, eight-in-ten reported engaging in informal supports, and one-in-ten did not engage in either. A majority of students strongly agreed (50.7%) or agreed (26.6%) that therapy or counseling would be helpful for their mental health. Encouragement from a friend to seek help was associated with significantly higher odds of therapy/counseling in the past 12 months (aOR = 4.89, 95% CI: 1.03–23.1). Encouragement from others—such as health professionals—was also associated with higher odds of antipsychotic medication use (aOR = 1.49, 95% CI: 1.01–2.20). Compared to students who believed that medication is very helpful for their mental health, students who identified medication (aOR=0.17, 95% CI: 0.09–0.31) or therapy/counseling as not helpful had lower odds of formal service use (aOR=0.31, 95% CI: 0.15–0.62).

**Conclusions:**

This is the largest known study of formal service and informal support engagement in students with a diagnosis of psychosis. Therapy and/or counseling in this population is highly utilized, and therapeutic resources available on college campuses may play an important role in supporting students diagnosed with psychosis. Encouragement from friends or healthcare professionals to seek help for mental health may promote help-seeking behaviors.

## Introduction

Psychosis is a severe mental illness that affects a small percentage of the population, with global lifetime prevalence estimates of 7.49 per 1,000 persons [1]. Despite its overall low prevalence, psychosis is a major public health concern largely due to delayed initiation of mental health care. In the United States, the median duration of untreated psychosis to initiation of first-episode psychosis programs is 74 weeks (nearly 1.5 years) [2]. Prolonged untreated symptoms of psychosis can lead to greater symptom severity, a lower likelihood of remission, poor treatment response, and diminished social functioning [3–5]. Early intervention and continued service provision play a crucial role in improving health outcomes and quality of life for individuals with psychosis [6, 7]. With the onset of psychosis typically emerging in young adulthood, and with evidence showing that early intervention leads to better health outcomes [7–9], understanding help-seeking behaviors and mental health service use among young adults with psychosis is a key priority.

There is substantial evidence linking medication non-adherence to high relapse rates in people experiencing psychosis, a major clinical concern in this population [10, 11]. Medication non-adherence is linked to increased emergency room visits, prolonged hospital stays, higher risks of suicidal ideation, and greater risk of relapse [10, 11]. While the existing literature highlights poor outcomes and high relapse rates associated with low treatment adherence in individuals diagnosed with psychosis, there is a research gap in understanding the factors, such as informal supports, that influence help-seeking behaviors and mental health service utilization [13, 14].

Early intervention services reduce the risk of treatment discontinuation and psychiatric hospitalization while improving outcomes related to overall quality of life, school or work involvement, symptom severity, and employment rates and relapse rates [7, 8]. The efficacy of treatment when initiated early and adhered to for individuals with psychosis motivates a need to examine factors that encourage treatment initiation [15]. Examining formal and informal health services utilization is especially important for the nearly 15 million college students in U.S. higher education [16]. This is particularly crucial as the traditional college years are characterized by young adults’ increasing independence in their health decision-making, including their use of health services. The traditional college years are an epidemiologically vulnerable time that coincides with the onset of many mental illnesses, including psychosis [9]. The average age of onset of psychosis (20.5 years) raises important questions about how U.S. college students with a diagnosis of psychosis access and navigate mental health services [9]. Many U.S. higher education institutions have rich resources to promote student well-being through organized social activities, counseling, peer support services, and or referrals [17, 18]. Exploring how college students diagnosed with psychosis engage with formal and informal mental health supports can help identify patterns of service utilization and elucidate opportunities for potential earlier intervention to improve psychosis outcomes.

This study seeks to examine the perceptions, beliefs, and attitudes that inhibit or facilitate formal service use and informal engagement (from friends, loved ones, roommates, campus staff, religious counselors, or support groups) among college students with a diagnosis of psychosis. Addressing the major knowledge gaps in mental health service utilization is a crucial first step toward reducing treatment delays that can improve outcomes for individuals with psychosis.

## Methods

### Data Source and Participants

This study utilizes the national Healthy Minds Study (HMS) cross-sectional data from 2015 to 2024 to examine mental health help-seeking among college students with a diagnosis of psychosis. HMS is an annual web-based survey that collects demographic information, assesses mental health status, and measures help-seeking behaviors among U.S. college students. All postsecondary institutions in the United States are eligible to participate in the HMS. From each participating university, students are randomly invited to complete HMS. Students are over 18 years old and consent to complete the survey, which is collected via Qualtrics and is IRB-approved. The response rates among students by survey year were as follows: 23% (2015), 27% (2016), 23% (2017) 16% (2018), 16% (2019), 14% (Fall 2020), 13% (Winter 2020), 15% (2021), 12% (2022), 9% (2023), and 9% (2024). As described below, survey nonresponse rates were applied to address some limitations related to response rates. Previous research has published extensively on HMS data collection methods (Lipson et al., 2022).

Diagnosis of psychosis was determined by the HMS survey item, *“Have you ever been diagnosed with any of the following conditions by a health professional?”(Select all that apply)*. Students were included in the sample if they selected the response option “Psychosis”.

## Measures

### Primary Outcomes

Formal mental health service utilization was categorized as participants engaging in therapy and/or antipsychotic medication in the past 12 months. Therapy utilization in the past 12 months was determined by the survey item *“How many total visits or sessions for counseling or therapy have you had in the past 12 months?”*. If students indicated that they had at least one therapy or counseling session in the past 12 months, then they were classified as ‘yes’ for therapy/counseling use, and if they did not, they were classified as ‘no’. Antipsychotic medication use was measured by the survey item “*In the past 12 months, have you taken any of the following types of prescription medications?*” *(Select all that apply)*. If students selected “Antipsychotics,” then they were classified as ‘yes’ for medication use, and if they did not, they were classified as ‘no’. Students were classified as 'yes' for formal service use if they had taken antipsychotic medication and/or attended therapy or counseling in the past 12 months; otherwise, they were classified as 'no'. Antipsychotic and therapy/counseling use in the past 12 months were also assessed as independent outcomes, but only among help seekers who engaged in formal service use due to survey skip logic.

Informal support engagement was determined by the survey question, “*In the past 12 months, have you received support for your mental or emotional health from any of the following sources?*” *(Select all that apply)*. Students were able to select “Roommate”, “Friend”, “Significant other”, “Family member”, “Religious counselor or other religious contact”, “Support group”, or “Other non-clinical sources, such as professor or staff member”. If any of those response options were selected, students were assigned a ‘yes’ to indicate informal support engagement, which was coded as a binary yes/no variable.

### Predictors

Reasons for seeking help were measured by the item, “*Earlier in this survey, you reported that you have taken medication and/or received counseling/therapy in the past 12 months for your mental or emotional health. Which of the following are important reasons why you received those services*?”. Students could select all that apply from the following reasons or factors that led them to seek care: “Self-initiated”, “Encouragement from family members”, “Pressure from family members”, “Encouragement from friends”, “Pressure from friends”, “Mandated or referred by a campus advisor”, “Someone other than a family member or friend, such as a health professional recommended or referred me to seek help”, and/or “Other”.

Perceived need for help was captured by survey item “*How much do you agree with the following statement? In the past 12 months, I needed help for emotional or mental health problems or challenges such as feeling sad, blue, anxious or nervous”*. Students were able to select “Strongly agree”, “Agree”, “Somewhat agree”, “Somewhat disagree”, “Disagree”, or “Strongly disagree”. Responses were coded numerically from 1 (“Strongly agree”) to 6 (“Strongly disagree”).

Perception of how helpful medication is was determined by the item “*How helpful on average do you think the medication would be for you if you were having mental or emotional health problems*?”. Students could select “Very helpful”, “Helpful”, “Somewhat helpful”, or “Not helpful”. Responses were coded numerically from 1 (“Very helpful”) to 4 (“Not helpful”).

Perception of how helpful therapy or counseling was measured by the item “*How helpful on average do you think therapy or counseling would be for you if you were having mental or emotional health problems?*. Students could select “Very helpful”, “Helpful”, “Somewhat helpful”, or “Not helpful”. Responses were coded numerically from 1 (“Very helpful”) to 4 (“Not helpful”).

### Statistical Analysis

Using administrative data provided to the HMS team, survey non-response weights were applied to all analyses to account for the higher female response rate than males to more accurately represent each university's female-male ratio. The weighted percent of participants’ age category, sex at birth, race, reasons for seeking help, perceived need for help, antipsychotic medication utilization in the past 12 months, therapy utilization in the past 12 months, perception of how helpful medication is, perception of how helpful therapy is, and survey year were examined by the total participant sample, formal, informal service use engagement, and no utilization in both formal and informal services.

While some individuals may have used both formal and informal supports, the primary focus of the analysis was to examine two sets of independent binary comparisons (1) students with a diagnosis of psychosis who report/do not report formal service use, and (2) students with a diagnosis of psychosis who report/do not report informal supports, rather than mutually exclusive service categories (e.g., informal only, formal only, both, or neither). Logistic regression models with coefficients exponentiated to adjusted odds ratios (aOR) were used to assess the probability of formal service use and informal engagement in students with a diagnosis of psychosis. Logistic regression models were also used to estimate aORs for antipsychotic and therapy/counseling engagement among help-seeking students who used formal mental health services. Predictors were chosen based on prior literature indicating a research gap on promotive factors improving medication and treatment adherence, low service utilization, and poor perception of treatment effectiveness in this population [19]. All models were adjusted for sex at birth (male and female), race (White, Asian, Hispanic/Latin(x), African American/ Black, or Other) and age categories (18–21, 22–25, 26–30, 31+). Analyses used a complete case approach, such that students with missing data on any variable included in the models were excluded from that analysis. All analyses were conducted using RStudio version 4.4.2.

## Results

### Characteristics of the Sample (Table 1)

Our analytic sample included 2,819 students from 665 colleges and universities. Of all students with psychosis, 81.9% reported engaging with formal service use, 76.7% reported engaging with informal supports, and 6% reported engagement in neither. Within this sample, just under half were between 18 and 21 years of age (43.1%), slightly over half were male (52.2%), and nearly six-in-ten were white (57.8%). Complete demographic data by formal and informal service-use engagement are reported in Table 1.

### Characteristics Related to Service Utilization

Within the last 12 months, 44.3% of students with a history of psychosis used antipsychotic medications and 82.4% engaged in therapy or counseling in the past 12 months. A total of 39.6% of students reported engaging in both antipsychotic medication and therapy or counseling in the past 12 months. Of students who reported informal support engagement, 11.9% of students did not have any formal service use in the past 12 months.

Among students engaged in formal services, 67.5% of students reported self-initiating or deciding to seek help on their own as an important reason for seeking medication and/or therapy/counseling. A majority (59%) of students with a diagnosis of psychosis strongly agreed that they needed help for emotional or mental health problems in the past 12 months. Most students (62.2%) believed that medication would be very helpful (34.0%) or helpful (28.2%), compared to somewhat helpful (19.4.%) or not helpful (18.4%) for their mental health. Similarly, a majority of students with a diagnosis of psychosis strongly agreed (50.7%) or agreed (26.6%) that therapy or counseling would be very helpful for them if they were having mental or emotional health problems, while a minority (15.1%) disagreed or (7.6%) strongly disagreed. A complete description of student characteristics related to service utilization is reported in Table 1.

#### Multivariable Results for Formal Service Use Utilization (Table 2)

Perceptions of need were strongly correlated with formal mental health service use among students with psychosis. Relative to students who strongly agreed that they needed help for their mental health, students who agreed that they needed help had 47% lower odds of engaging in formal service use (aOR = 0.53, 95% CI 0.32–0.86) while students who somewhat agreed that they needed help had 63% lower odds of formal service use (aOR = 0.37, 95% CI 0.22–0.63). Relative to students who strongly agreed that they needed help for their mental health, students who somewhat disagreed (aOR = 0.14, 95% CI 0.07–0.29) or disagreed (aOR = 0.14, 95% CI 0.07–0.28) had 86% lower odds of formal service use engagement. Compared to students who strongly agreed that they needed help for their mental health, students who strongly disagreed had 70% lower odds of formal service use engagement (aOR = 0.30, 95% CI 0.15–0.60).

Student perceptions of how helpful medication would be for them was significantly associated with formal service use. Compared to students who believed medication would be very helpful for their mental health, students who believed medication would not be helpful had 83% lower odds of engaging in formal services (aOR = 0.17, 95% CI: 0.09–0.31).

Similarly, student perceptions of how helpful therapy would be for them was associated with engagement in formal service use. Relative to students who believed that therapy would be very helpful for their mental health, students who identified therapy as not helpful for their mental health had 69% lower odds of engaging in formal service (aOR = 0.31, 95% CI 0.15–0.62).

#### Multivariable Results for Types of Supports Associated with Past-Year Antipsychotic Medication and Therapy/Counseling Utilization Among Students Engaged in Formal Service Use (Table 3)

Among help-seeking students engaged in formal services, encouragement from someone other than a friend or family member (such as a health professional) to seek help for mental health was also associated with antipsychotic use in the past 12 months. Relative to those who did not receive encouragement from someone other than a friend or family member, students who received encouragement to seek help (from someone other than a friend or family member, such as a health professional) had 1.49 times higher odds of taking antipsychotic medication in the past 12 months (95% CI 1.01–2.20). Encouragement from a friend to seek help for mental health was associated with therapy/counseling in the past 12 months. Relative to those who did not receive encouragement from friends to seek help, students who did had 4.89 times higher odds of therapy/counseling engagement in the past 12 months (95% CI 1.03–23.1).

### Multivariable Results for Informal Service Use Utilization (Table 2)

Perceived need for help was associated with informal service use. Relative to students who strongly agreed that they needed help for emotional or mental health problems or challenges, students who somewhat agreed had 41% lower odds of engaging in informal supports (aOR = 0.59, 95% CI 0.35–0.99). Similarly, relative to students who strongly agreed to needing help for their mental health, students who somewhat disagreed had 57% lower odds of engaging in informal support (aOR = 0.43, 95% CI 0.22–0.87). Student perceptions of how helpful medication or therapy would be for their mental health was not associated with engagement in informal service use.

## Discussion

This study examined the reasons why students with a diagnosis of psychosis seek help, their perceived need for help, and their perception of how effective medication and or therapy would be for their mental health, as predictors of formal service and informal support engagement using national HMS data from 2015–2024. This is the largest known study of formal and informal service utilization in students with a diagnosis of psychosis. The help-seeking behaviors of individuals with psychosis remains largely underexplored in the existing literature, particularly with larger samples.

This study of 2,819 students diagnosed with psychosis found that four-in-ten students report taking an antipsychotic medication in the past 12 months and eight-in-ten report utilizing counseling/therapy in the past 12 months, reflecting high rates of mental health service utilization. A total of four-in-ten students engaged in both antipsychotic medication and counseling/therapy in the past 12 months. This means—six-in-ten students are not meeting the recommended combination of both pharmacological and psychosocial treatment standard of care guidelines [20]. Negative perceptions of how helpful medication and or therapy/counseling would be for mental health was associated with significantly lower odds of engagement in formal service use. Knowledge of symptoms, as well as the efficacy of medication and therapy or counseling to promote treatment seeking, could be targeted through psychoeducation, but needs to be further explored [21].

Studies have been able to capture antipsychotic prescribing trends in claims data [22, 23], but often fail to capture or take into account medication non-adherence rates which can be as high as 60% in this population [24], making the student self-reported medication and therapy or counseling in the past 12 months a unique comparator to prior research. Despite the high rates of medication non-adherence [24] and the strong engagement of therapy or counseling in the past 12 months, there remains an underutilized potential to leverage therapeutic interventions within this population. Roughly four-in-five students with psychosis agreed that they needed help for emotional or mental health problems in the past 12 months. This further underscores a potentially unmet need in this population and highlights an opportunity to employ therapeutic interventions that are highly receptive in this population. More than three-in-four students with psychosis strongly agreed or agreed that therapy or counseling would be very helpful for them if they were having mental or emotional health problems. Universities and clinicians alike can continue to capitalize on this population's acceptability and strong engagement towards therapeutic interventions, which have been shown to reduce relapse, improve quality of life, and psychotic symptoms [25–27].

Mental health outcomes and service engagement are poorly understood when treatment is self-initiated, encouraged, vs mandated in individuals with psychosis. Overall, we found that those who are encouraged by friends or health professionals versus pressured or mandated as primary reasons for seeking help have higher odds of antipsychotic and or therapy/counseling use. This is an important implication given the institutionalization of mental illness that can result in mandated treatment [28]. Support systems, such as friends, play a crucial role in identifying early psychosis symptoms and help navigate mental health services, which may be an important factor in treatment initiation [29–31]. Future research, particularly longitudinal studies, should explore the long-term outcomes of therapy and antipsychotic engagement in individuals who are encouraged, pressured, or mandated to treatment.

We found that lack of perceived need for help was associated with significantly lower odds of formal service use in this sample. While acknowledgment or insight of mental illness may affect treatment-seeking and adherence [32], frameworks such as subjective illness theory and self-determination theory can help illuminate the help-seeking behaviors among individuals with psychosis. Subjective illness theory emphasizes how individuals diagnosed with psychosis view their illness, including its attributed cause, and outlook of prognosis differently based on environmental or personally held beliefs to cope or make meaning of their illness, which can affect how individuals engage with services [33]. Other models, such as self-determination theory, stress the importance of an individual's motivation, which influences treatment-seeking behaviors [34]. While frameworks may provide some insight into treatment-seeking, future scholarship should continue to refine frameworks that elucidate treatment-seeking behaviors, which is crucial due to the severely delayed initiation of care in individuals with psychosis [2, 5].

The present study also examined the informal supports of students as an important resource affecting outcomes that is often not quantified or measured in studies. We found that the perceived need for help was also associated with informal engagement. Relative to students that strongly agreed that they needed help for emotional or mental health challenges, students who somewhat agreed or disagreed had significantly lower odds of engaging in informal supports. Support from friends, family, significant others, religious counselors, support groups, or campus may play a key role in supporting students' overall well-being as well as treatment initiation. Informal supports, such as religious or spiritual healers, are often the first initial source of help in the initiation of symptoms, and are well accepted within this population [35]. In college and university settings, informal services and supports often come in the form of peer (i.e., student-to-student) or support groups. Peer support services promote positive vocational outcomes in first-episode psychosis programs, including higher odds of finding employment or enrolling in education [36].

Even though this is the largest study of formal service and informal support engagement in students with psychosis, this study has limitations. While this study draws on national data from 2015 to 2024, this is a cross-sectional study and students were not followed over time. We therefore cannot establish causality. With these survey data, it is not possible to determine the severity of their condition or whether students experienced multiple episodes of psychosis, a common attribute of the condition that may affect formal service and informal support engagement [10, 11, 38]. Furthermore, we do not know the quality or intensity of the services students received. While students were randomly invited to complete the survey, students who completed the survey could have inherent characteristic differences between students who completed the survey and those who did not. For instance, because psychosis diagnoses were self-reported, there is an inability to know whether students with psychosis were more or less likely to respond to the survey. Secondly, while this study controlled for student characteristics including race, sex at birth, and age – residual confounding is a concern. For example, important characteristics such as the date of the last psychotic episode were not measured and are therefore missing from the model.

## Conclusions

This is the largest known study of formal and informal support engagement in students with a diagnosis of psychosis. Therapy and/or counseling in this population is highly utilized and identified as being helpful for students' mental health if they were having mental health challenges. As a result, therapeutic services on college campuses play an important role as an identified resource to college students with psychosis. Most students also identified that they needed help for emotional or mental health problems in the past 12 months. Psychoeducation could target perceptions of how effective or helpful medication or therapy/counseling is for mental health, which could facilitate help-seeking behaviors for formal and informal support engagement, but needs to be further explored. Lastly, encouragement from friends and or health professionals to seek treatment for mental health may promote help-seeking behaviors. Future research, particularly longitudinal studies, should try to distinguish the long-term mental health and service engagement outcomes when individuals are encouraged, pressured, or mandated to treatment.

## Figures and Tables

**Table 1:** Characteristics of the analytic sample of participants from the 2015–2024 waves of the Healthy Minds Study with self-reported psychosis diagnosis (n = 2819)

	All Students with Psychosis	Students with Formal service use engagement	Students with Informal service use engagement	Students with no formal and informal engagement
Unweighted Sample Size	N = 2819	N = 2,310	N = 2,163	N = 171
	Weighted %	Weighted %	Weighted %	Weighted %
**Age Category**				
18–21	43.1	44.0	42.7	45.2
22–25	20.0	20.7	21.9	23.9
26–30	14.2	12.3	14.2	13.1
31+	21.8	23.1	21.2	17.8
**Sex**				
Male	52.2	53.7	54.8	43.8
Female	46.9	45.5	44.1	55.8
Intersex	0.9	0.9	1.0	0.4
**Race**				
White	57.8	58.2	58.9	52.2
Hispanic/Latin(x)	11.3	11.2	11.8	10.0
African American/ Black	13.7	13.8	12.7	10.9
Asian	5.5	5.1	5.9	9.3
Middle Eastern, Arab, or Arab American	1.9	2.1	1.7	1.1
Native Hawaiian / Alaskan / or Pacific Islander	4.6	4.6	4.9	7.4
Other	5.2	5.0	4.1	9.1
**Survey year**				
2015	1.6	1.2	1.6	3.5
2016	0.6	0.5	0.7	0.3
2017	3.7	3.5	3.9	5.3
2018	6.2	6.1	6.8	7.7
2019	6.9	6.7	6.9	7.9
2020	5.9	5.8	5.5	8.5
2021	12.4	12.0	12.1	14.7
2022	21.4	20.8	22.1	13.2
2023	21.5	21.9	22.0	26.0
2024	19.6	21.6	18.5	12.8
**Reasons for seeking help**				
Decided to seek help on own	-	67.5	-	-
A friend encouraged me to seek help	-	17.1	-	-
A friend pressured me to seek help	-	6.6	-	-
A family member encouraged me to seek help	-	30.5	-	-
A family member pressured me to seek help	-	14.7	-	-
Someone other than a friend or family member encouraged me to seek help, such as a health professional	-	28.2	-	-
Campus advisor mandated or referred me to seek help	-	6.1	-	-
I inquired more information about my options	-	1.2	-	-
Other	-	7.2	-	-
**Perceived need for help**				
Strongly Agree	59.0	63.4	62.3	34.5
Agree	18.5	18.0	17.4	19.2
Somewhat Agree	9.8	9.0	8.7	13.1
Somewhat Disagree	2.4	1.6	2.0	5.9
Disagree	5.0	3.4	4.8	11.1
Strongly Disagree	5.3	4.6	4.8	16.2
**Antipsychotic Utilization in the past 12 months**				
No Antipsychotic	55.7	45.7	53.9	100
Antipsychotic	44.3	54.3	46.1	0
**Therapy Utilization in the past 12 months**				
No Therapy	17.6	4.2	17.4	100
Therapy	82.4	95.8	82.6	0
**Both Therapy and Antipsychotic Utilization in the past 12 months**	39.6	48.6	42.0	0
**Perception of how helpful medication is**				
Very Helpful	34.0	37.2	35.4	20.1
Helpful	28.2	29.2	27.3	27.8
Somewhat Helpful	19.4	20.6	19.4	15.0
Not Helpful	18.4	12.9	17.9	37.2
**Perception of how helpful therapy is**				
Very Helpful	50.7	52.0	52.4	43.0
Helpful	26.6	27.1	26.3	22.0
Somewhat Helpful	15.1	14.9	14.7	18.4
Not Helpful	7.6	6.0	6.7	16.7

Note: Reasons for seeking help could not be calculated for all students, students with Informal service use engagement, and students with no formal/informal engagement due to survey skip logic

**Table 2:** Adjusted logistic regression models assessing the associations between participant beliefs and use of formal and informal services among participants with self-reported psychosis diagnoses in the 2015–2024 Healthy Minds Study

	Unweighted n in the total sample	OR Formal Service Use	95% CI	p value	OR Informal Service Use	95% CI	p value
**Perceived Need for Help** ^a,d^	2,795						
Strongly Agree	1,730	ref			ref		
Agree	499	**0.53**	**(0.32, 0.86)**	**0.011**	0.66	(0.41, 1.04)	0.073
Somewhat Agree	242	**0.37**	**(0.22, 0.63)**	**< 0.001**	**0.59**	**(0.35, 0.99)**	**0.049**
Somewhat Disagree	77	**0.14**	**(0.07, 0.29)**	**< 0.001**	**0.43**	**(0.22, 0.87)**	**0.018**
Disagree	119	**0.14**	**(0.07, 0.28)**	**< 0.001**	0.83	(0.43, 1.6)	0.581
Strongly Disagree	128	**0.30**	**(0.15, 0.60)**	**0.001**	0.59	(0.31, 1.1)	0.101
**Perceptions of How Helpful Medication is** ^b,e^	1,345						
Very Helpful	488	ref			ref		
Helpful	368	0.67	(0.37, 1.26)	0.215	0.74	(0.41, 1.32)	0.307
Somewhat Helpful	263	0.73	(0.39, 1.4)	0.337	0.90	(0.50, 1.63)	0.733
Not Helpful	226	**0.17**	**(0.09, 0.31)**	**< 0.001**	0.89	(0.47, 1.70)	0.725
**Perception of How Helpful Therapy Is** ^c,f^	1,345						
Very Helpful	657	ref			ref		
Helpful	372	0.90	(0.47, 1.72)	0.758	0.75	(0.41, 1.36)	0.834
Somewhat Helpful	202	0.76	(0.39, 1.47)	0.412	0.711	(0.39, 1.27	0.250
Not Helpful	113	**0.31**	**(0.15, 0.62)**	**0.001**	0.51	(0.26, 1.01)	0.052

Note: Significant associations are in bold. All models adjusted for race (Asian, Latin(x)/Hispanic, African American/ Black, Other), Sex at birth (Male and Female), and age categories (18–21, 22–25, 26–30, 31+).

Footnotes:

a Model a assessed the association between the perceived need for help and formal service use engagement, adjusting for the same covariates (n = 1,325).

b Model b assessed the association between the perceived helpfulness of medication and formal service use engagement (n = 1,324).

c Model b assessed the association between the perceived helpfulness of therapy and formal service use engagement (n = 1,463).

d Model d assessed the association between the perceived need for help and informal support engagement (n = 2,756).

e Model e assessed the association between the perceived helpfulness of medication and informal support engagement (n = 1,325).

f Model f assessed the association between the perceived helpfulness of therapy and informal support engagement (n = 1,324)

**Table 3:** Adjusted logistic regression models assessing the associations between reasons for seeking help and antipsychotic and or therapy/counseling use in the past 12 months among students engaged in formal services with self-reported psychosis diagnoses in the 2015–2024 Healthy Minds Study

	Unweighted n in the total sample	OR Antipsychotic Use	95% CI	p value	OR Therapy/Counseling	95% CI	p value
**Reasons for Seeking Help** ^g,h^	1,734						
Decided to seek help on own	1,276	0.79	(0.52, 1.20)	0.268	1.81	(0.77, 4.27)	0.170
A friend encouraged me to seek help	319	0.96	(0.61, 1.50)	0.855	**4.89**	**(1.03, 23.1)**	**0.045**
A friend pressured me to seek help	126	1.00	(0.48, 2.08)	0.991	0.65	(0.16, 3.1)	0.634
A family member encouraged me to seek help	557	1.05	(0.72, 1.54)	0.803	1.23	(0.53, 2.86)	0.637
A family member pressured me to seek help	296	1.12	(0.70, 1.78)	0.635	0.84	(0.34, 2.03)	0.693
Someone other than a friend or family member encouraged me to seek help, such as a health professional	429	**1.49**	**(1.01, 2.20)**	**0.044**	1.26	(0.45, 3.51)	0.659
Campus advisor mandated or referred me to seek help	115	1.11	(0.61, 2.01)	0.732	0.40	(1.07, 1.48)	0.659
I inquired more information about my options	25	0.62	(0.18, 2.11)	0.442	-	-	-
Other	104	0.80	(0.43, 1.51)	0.493	0.71	(0.17, 2.89)	0.632

Note: Significant associations are in bold. All models adjusted for race (Asian, Latin(x)/Hispanic, African American/ Black, Other), Sex at birth (Male and Female), and age categories (18–21, 22–25, 26–30, 31+).

Footnotes:

g Model g assessed the association between reasons for seeking help and antipsychotic use in the past 12 months (n = 1,724)

h Model h assessed the association between the reasons for seeking help and therapy/counseling in the past 12 months (n = 1,691)

## References

[1] Moreno-Küstner B, Martín C, Pastor L (2018) Prevalence of psychotic disorders and its association with methodological issues. A systematic review and meta-analyses. PLoS ONE 13(4):e0195687. 10.1371/journal.pone.0195687 29649252 PMC5896987

[2] Addington J, Heinssen RK, Robinson DG, Schooler NR, Marcy P, Brunette MF, Correll CU, Estroff S, Mueser KT, Penn D, Robinson JA, Rosenheck RA, Azrin ST, Goldstein AB, Severe J, Kane JM (2015) Duration of Untreated Psychosis in Community Treatment Settings in the United States. Psychiatric Serv 66(7):753–756. 10.1176/appi.ps.201400124 25588418

[3] Drake RJ, Husain N, Marshall M, Lewis SW, Tomenson B, Chaudhry IB, Everard L, Singh S, Freemantle N, Fowler D, Jones PB, Amos T, Sharma V, Green CD, Fisher H, Murray RM, Wykes T, Buchan I, Birchwood M (2020) Effect of delaying treatment of first-episode psychosis on symptoms and social outcomes: A longitudinal analysis and modelling study. Lancet Psychiatry 7(7):602–610. 10.1016/S2215-0366(20)30147-4 32563307 PMC7606908

[4] Fresan A, Apiquian R, Robles-García R, Zarate C-AT, Balducci PM, Broussard B, Wan CR, Compton MT (2020) Similarities and Differences in Associations Between Duration of Untreated Psychosis (DUP) and Demographic, Premorbid, and Symptom Severity Measures in Two Samples of First-Episode Psychosis Patients from Mexico and the United States. Psychiatr Q 91(3):769–781. 10.1007/s11126-020-09736-3 32221766 PMC7780290

[5] Penttilä M, Jääskeläinen E, Hirvonen N, Isohanni M, Miettunen J (2014) Duration of untreated psychosis as predictor of long-term outcome in schizophrenia: Systematic review and meta-analysis. Br J Psychiatry 205(2):88–94. 10.1192/bjp.bp.113.127753 25252316

[6] Chan SKW, Chan HYV, Devlin J, Bastiampillai T, Mohan T, Hui CLM, Chang WC, Lee EHM, Chen EYH (2019) A systematic review of long-term outcomes of patients with psychosis who received early intervention services. Int Rev Psychiatry 31(5–6):425–440. 10.1080/09540261.2019.1643704 31353981

[7] Correll CU, Galling B, Pawar A, Krivko A, Bonetto C, Ruggeri M, Craig TJ, Nordentoft M, Srihari VH, Guloksuz S, Hui CLM, Chen EYH, Valencia M, Juarez F, Robinson DG, Schooler NR, Brunette MF, Mueser KT, Rosenheck RA, Kane JM (2018) Comparison of Early Intervention Services vs Treatment as Usual for Early-Phase Psychosis: A Systematic Review, Meta-analysis, and Meta-regression. JAMA Psychiatry 75(6):555–565. 10.1001/jamapsychiatry.2018.0623 29800949 PMC6137532

[8] de Salazar G, Guinart D, Armendariz A, Aymerich C, Catalan A, Alameda L, Rogdaki M, Martinez Baringo E, Soler-Vidal J, Oliver D, Rubio JM, Arango C, Kane JM, Fusar-Poli P, Correll CU (2024) Duration of Untreated Psychosis and Outcomes in First-Episode Psychosis: Systematic Review and Meta-analysis of Early Detection and Intervention Strategies. Schizophr Bull 50(4):771–783. 10.1093/schbul/sbae017 38491933 PMC11283197

[9] Solmi M, Radua J, Olivola M, Croce E, Soardo L, Salazar de Pablo G, Il Shin J, Kirkbride JB, Jones P, Kim JH, Kim JY, Carvalho AF, Seeman MV, Correll CU, Fusar-Poli P (2022) Age at onset of mental disorders worldwide: Large-scale meta-analysis of 192 epidemiological studies. Mol Psychiatry 27(1):281–295. 10.1038/s41380-021-01161-7 34079068 PMC8960395

[10] Caseiro O, Pérez-Iglesias R, Mata I, Martínez-Garcia O, Pelayo-Terán JM, Tabares-Seisdedos R, de la Ortiz-García V, Vázquez-Barquero JL, Crespo-Facorro B (2012) Predicting relapse after a first episode of non-affective psychosis: A three-year follow-up study. J Psychiatr Res 46(8):1099–1105. 10.1016/j.jpsychires.2012.05.001 22721546

[11] Robinson D, Woerner MG, Alvir JMJ, Bilder R, Goldman R, Geisler S, Koreen A, Sheitman B, Chakos M, Mayerhoff D, Lieberman JA (1999a) Predictors of Relapse Following Response From a First Episode of Schizophrenia or Schizoaffective Disorder. Arch Gen Psychiatry 56(3):241–247. 10.1001/archpsyc.56.3.241 10078501

[12] Higashi K, Medic G, Littlewood KJ, Diez T, Granström O, De Hert M (2013) Medication adherence in schizophrenia: Factors influencing adherence and consequences of nonadherence, a systematic literature review. Therapeutic Adv Psychopharmacol 3(4):200–218. 10.1177/2045125312474019 PMC3805432 24167693

[13] Oliver D, Davies C, Crossland G, Lim S, Gifford G, McGuire P, Fusar-Poli P (2018) Can We Reduce the Duration of Untreated Psychosis? A Systematic Review and Meta-Analysis of Controlled Interventional Studies. Schizophr Bull 44(6):1362–1372. 10.1093/schbul/sbx166 29373755 PMC6192469

[14] Smolak A, Gearing RE, Alonzo D, Baldwin S, Harmon S, McHugh K (2013) Social Support and Religion: Mental Health Service Use and Treatment of Schizophrenia. Commun Ment Health J 49(4):444–450. 10.1007/s10597-012-9536-8 PMC3570737 22855264

[15] Kelebie M, Kibralew G, Tadesse G, Rtbey G, Aderaw M, Endeshaw W, Belachew M, Muche M, Getnet D, Fentahun S (2025) Effectiveness of antipsychotic medication in patients with schizophrenia in a real world retrospective observational study in Ethiopia. Sci Rep 15(1):4663. 10.1038/s41598-025-85832-3 39920141 PMC11806019

[16] National Center for Education Statistics (2013) The NCES Fast Facts Tool provides quick answers to many education questions. Retrieved March 3, 2025, from https://nces.ed.gov/fastfacts/display.asp?id=98

[17] Downs N, Galles E, Skehan B, Lipson SK (2018) Be True to Our Schools—Models of Care in College Mental Health. Curr Psychiatry Rep 20(9):1–8. 10.1007/s11920-018-0935-6 30094519

[18] Lipson SK, Zhou, Sasha WIII, Blake, Beck, Katie,and, Eisenberg D (2016) Major Differences: Variations in Undergraduate and Graduate Student Mental Health and Treatment Utilization Across Academic Disciplines. Journal of College Student Psychotherapy, 30(1), 23–41. 10.1080/87568225.2016.1105657

[19] Curto M, Fazio, Francesco U (2021),Martina, Navari, Serena, Lionetto, Luana, & and Baldessarini, R. J. Improving adherence to pharmacological treatment for schizophrenia: A systematic assessment. Expert Opinion on Pharmacotherapy, 22(9), 1143–1155. 10.1080/14656566.2021.1882996 33543659

[20] American Psychiatric Association (2020) The American Psychiatric Association Practice Guideline for the Treatment of Patients With Schizophrenia. American Psychiatric Association Publishing. 10.1176/appi.books.9780890424841

[21] Herrera SN, Sarac C, Phili A, Gorman J, Martin L, Lyallpuri R, Dobbs MF, DeLuca JS, Mueser KT, Wyka KE, Yang LH, Landa Y, Corcoran CM (2023) Psychoeducation for individuals at clinical high risk for psychosis: A scoping review. Schizophr Res 252:148–158. 10.1016/j.schres.2023.01.008 36652831 PMC9974813

[22] Li P, Benson C, Geng Z, Seo S, Patel C, Doshi JA (2023) Antipsychotic utilization, healthcare resource use and costs, and quality of care among fee-for-service Medicare beneficiaries with schizophrenia in the United States. J Med Econ 26(1):525–536. 10.1080/13696998.2023.2189859 36961119

[23] Huang A, Amos TB, Joshi K, Wang L, Nash A (2018) Understanding healthcare burden and treatment patterns among young adults with schizophrenia. J Med Econ 21(10):1026–1035. 10.1080/13696998.2018.1500370 30001651

[24] Offord S, Lin J, Mirski D, Wong B (2013) Impact of early nonadherence to oral antipsychotics on clinical and economic outcomes among patients with schizophrenia. Adv Therapy 30(3):286–297. 10.1007/s12325-013-0016-5 23483449

[25] Lutgens D, Gariepy G, Malla A (2017) Psychological and psychosocial interventions for negative symptoms in psychosis: Systematic review and meta-analysis. Br J Psychiatry 210(5):324–332. 10.1192/bjp.bp.116.197103 28302699

[26] Solmi M, Croatto G, Piva G, Rosson S, Fusar-Poli P, Rubio JM, Carvalho AF, Vieta E, Arango C, DeTore NR, Eberlin ES, Mueser KT, Correll CU (2023) Efficacy and acceptability of psychosocial interventions in schizophrenia: Systematic overview and quality appraisal of the meta-analytic evidence. Mol Psychiatry 28(1):354–368. 10.1038/s41380-022-01727-z 35999275

[27] Yıldız E (2020) The effects of acceptance and commitment therapy in psychosis treatment: A systematic review of randomized controlled trials. Perspect Psychiatr Care 56(1):149–167. 10.1111/ppc.12396 31074039

[28] Plahouras JE, Mehta S, Buchman DZ, Foussias G, Daskalakis ZJ, Blumberger DM (2020) Experiences with legally mandated treatment in patients with schizophrenia: A systematic review of qualitative studies. Eur Psychiatry 63(1):e39. 10.1192/j.eurpsy.2020.37 32406364 PMC7355163

[29] Causier C, Waite F, Sivarajah N, Knight MTD (2024) Structural barriers to help-seeking in first-episode psychosis: A systematic review and thematic synthesis. Early Interv Psychiat 18(5):293–311. 10.1111/eip.13497 38356356

[30] Harandi TF, Taghinasab MM, Nayeri TD (2017) The correlation of social support with mental health: A meta-analysis. Electron Physician 9(9):5212–5222. 10.19082/5212 29038699 PMC5633215

[31] Topor A, Borg M, Mezzina R, Sells D, Marin I, Davidson L (2006) Others: The role of family, friends, and professionals in the recovery process. Am J Psychiatric Rehabilitation 9(1):17–37. 10.1080/15487760500339410

[32] Lincoln TM, Lüllmann E, Rief W (2007) Correlates and Long-Term Consequences of Poor Insight in Patients With Schizophrenia. Syst Rev Schizophrenia Bull 33(6):1324–1342. 10.1093/schbul/sbm002 PMC2779879 17289653

[33] Holzinger A, Löffler W, Müller P, Priebe S, Angermeyer MC (2002) SUBJECTIVE ILLNESS THEORY AND ANTIPSYCHOTIC MEDICATION COMPLIANCE BY PATIENTS WITH SCHIZOPHRENIA. J Nerv Ment Dis 190(9):597 12357093 10.1097/01.NMD.0000030524.45210.FD

[34] Jochems EC, Duivenvoorden HJ, van Dam A, van der Feltz-Cornelis CM, Mulder CL (2017) Motivation, treatment engagement and psychosocial outcomes in outpatients with severe mental illness: A test of Self-Determination Theory. Int J Methods Psychiatr Res 26(3):e1537. 10.1002/mpr.1537 27790822 PMC6877197

[35] Huguelet P, Mohr S, Betrisey C, Borras L, Gillieron C, Marie AM, Rieben I, Perroud N, Brandt P-YA randomized trial of spiritual assessment of outpatients with schizophrenia: Patients’ and clinicians’ experience. Psychiatric Services (, Washington DC (2011) 62(1), 79–86. 10.1176/ps.62.1.pss6201_0079 21209304

[36] Alvarez-Jimenez M., Koval P., Schmaal L., Bendall S., O’Sullivan S., Cagliarini D., D’Alfonso S., Rice S., Valentine L., Penn D. L., Miles C., Russon P., Phillips J., McEnery C., Lederman R., Killackey E., Mihalopoulos C., Gonzalez-Blanch C., Gilbertson T., … Gleeson J. F. M. (2021). The Horyzons project: A randomized controlled trial of a novel online social therapy to maintain treatment effects from specialist first-episode psychosis services. World Psychiatry, 20(2), 233–243. 10.1002/wps.20858 34002511 PMC8129860

[37] Brown E, Bedi G, McGorry P, O’Donoghue B (2020) Rates and Predictors of Relapse in First-Episode Psychosis: An Australian Cohort Study. Schizophrenia Bull Open 1(1):sgaa017. 10.1093/schizbullopen/sgaa017

